# Site-specific factors associated with clinical trial recruitment efficiency in general practice settings: a comparative descriptive analysis

**DOI:** 10.1186/s13063-023-07177-4

**Published:** 2023-03-04

**Authors:** Michelle Tew, Max Catchpool, John Furler, Katie De La Rue, Philip Clarke, Jo-Anne Manski-Nankervis, Kim Dalziel

**Affiliations:** 1 Centre for Health Policy, Melbourne School of Population and Global Health, The University of Melbourne, Parkville, VIC Australia; 2 Department of General Practice, University of Melbourne, 780 Elizabeth St, Melbourne, VIC Australia; 3 Health Economics Research Centre, Nuffield Department of Population Health, University of Oxford, Oxford, UK

**Keywords:** Recruitment efficiency, Cost, Clinical trial, Diabetes, General practice

## Abstract

**Background:**

Recruitment of participants is crucial to the success of randomised control trials (RCTs) but can be challenging and expensive. Current research on trial efficiency is often focused at the patient-level with an emphasis on effective recruitment strategies. Less is known about selection of study sites to optimise recruitment. We examine site-level factors that are associated with patient recruitment and cost efficiency using data from an RCT conducted across 25 general practices (GP) in Victoria, Australia.

**Methods:**

Data on number of participants screened, excluded, eligible, recruited, and randomised from each study site were extracted from a clinical trial. Details regarding site characteristics, recruitment practices, and staff time commitment were collected using a three-part survey. The key outcomes assessed were recruitment efficiency (ratio of screened to randomised), average time, and cost for each participant recruited and randomised. To identify practice-level factors associated with efficient recruitment and lower cost, outcomes were dichotomised (25th percentile vs others) and each practice-level factor assessed against the outcomes to determine its association.

**Results:**

Across 25 GP study sites, 1968 participants were screened of which 299 (15.2%) were recruited and randomised. The mean recruitment efficiency was 7.2, varying from 1.4 to 19.8 across sites. The strongest factor associated with efficiency was assigning clinical staff to identify potential participants (57.14% vs. 22.2%). The more efficient sites were smaller practices and were more likely to be rural locations and in areas of lower socioeconomic status. The average time used for recruitment was 3.7 h (SD2.4) per patient randomised. The mean cost per patient randomised was $277 (SD161), and this varied from $74 to $797 across sites. The sites identified with the 25% lowest recruitment cost (*n* = 7) were more experienced in research participation and had high levels of nurse and/or administrative support.

**Conclusion:**

Despite the small sample size, this study quantified the time and cost used to recruit patients and provides helpful indications of site-level characteristics that can help improve feasibility and efficiency of conducting RCT in GP settings. Characteristics indicative of high levels of support for research and rural practices, which often tends to be overlooked, were observed to be more efficient in recruiting.

**Supplementary Information:**

The online version contains supplementary material available at 10.1186/s13063-023-07177-4.

## Background

Randomised control trials (RCT) are the gold standard for studying causal relationships and produce the most reliable evidence about the benefits and harms of clinical interventions. Depending on the phase and focus of the trial, the cost of conducting an RCT can range from US$3.4 million to $21.4 million [1]. The success of the trial is reliant on being able to recruit the target number of participants to avoid being underpowered to statistically demonstrate the effectiveness of the intervention. It is well documented that costs associated with patient recruitment is an important driver of total RCT costs [1, 2]. Recruitment is a challenging and expensive endeavour with up to 50% of RCTs failing to recruit to target and only 25% successfully recruiting in a timely manner [3]. As such, problems with recruitment can be costly as a consequence of extending recruitment period or increasing the number of sites and sometimes result in needing to seek or supplement with additional funds [4]. Recruitment inefficiency also results in wasted resources (in both sunk and opportunity costs) if trials discontinue with insufficient patient numbers as well as the ethical consequences of failing to answer the clinical question posed and patients’ commitment [5]. Therefore, having effective strategies that optimise recruitment particularly in the planning stages is crucial.

Surprisingly, given the high cost of conducting clinical trials, there is limited published data on trial resource use and costs likely signifying the lack of transparent and comprehensive empirical data on RCT costs [6]. There is also a dearth of literature on recruitment efficiency. Most research have focused on improving the effectiveness of recruitment; for example, specific recruitment strategies to increase participant recruitment and retention [7–10] and clinician characteristics [3, 11]. Without guidance and empirical evidence on cost items for RCTs or an understanding of what contributes to running an efficient trial, trial investigators are often reliant on their past experience when planning their trials. Further, experiences reported from seven primary care-based clinical trials indicate that characteristics related to the sites such as previous experience of research were more influential in trial recruitment than details of specific interventions [12]. Therefore, study site selection is an important aspect of the clinical trial process with poor choices potentially leading to study failure or becoming expensive due to the inclusion of additional sites [3, 13].

Using data from an RCT conducted across Australian general practices, we describe the resources used for recruitment and evaluate the indicators for efficient recruitment. The main aims of this study were to determine the following:

1. Efficiency of recruitment, defined as the number of patients recruited and randomised relative to the numbers screened
2. Average time spent for each patient randomised
3. Average cost per patient randomised
4. Practice-level factors associated with more efficient recruitment and lower cost

## Methods

GP-OSMOTIC was a two-arm, open label, 12-month individually randomised controlled trial across 25 general practices in Victoria, Australia. The target population was adults (aged ≥ 18 years) with type 2 diabetes (T2D) whose HbA1c was above recommended target, despite prescription of at least two non-insulin hypoglycaemic agents and/or insulin. The intervention involved the use of a professional-mode flash glucose monitoring system and patients in the control arm received usual clinical care. The study design, its findings and cost-effectiveness have been reported in detail [14–16].

Practices were selected from a database of research and teaching active practices associated with the Department of General Practice at the University of Melbourne. Those with at least one consenting general practitioner are eligible to be part of the trial. Practices generated a list of potentially eligible people with T2D to be screened for full eligibility by searching their practice electronic medical record (EMR) database or using an audit tool. People with T2D were invited by letter and/or telephone call to attend the practice to hear more about the study and if interested to give consent. After consenting, a clinically trained research assistant (RA) collected baseline data and arranged baseline pathology tests. Study data collection and randomisation were managed using REDCap [17] electronic data capture tools. Consenting eligible participants were randomised (1:1) after the baseline flash glucose monitor was attached. Masking (of participants or their treating clinicians) to allocation was not possible. Data on the number of participants screened, excluded, eligible, and recruited (and randomised) from each of the study sites were compiled for analysis.

A questionnaire was prospectively developed to capture details relating to study site and recruitment and was completed for each study site. The questionnaire included three sections (Appendix 1 in Supplementary Material). The first section (A) contained questions about study site characteristics and was completed at the start of the trial by the practice manager. Section B contained questions on willingness to participate and identification of key individuals involved in recruitment completed by the study co-ordinator at the end of recruitment. Section C, completed by the practice manager or similar, reported the time commitment (hours) of various staff at the practice and was completed at the end of the recruitment period. Socioeconomic status were assigned to each site according to the Australian Bureau of Statistics (ABS) Index of Relative Socio-economic Advantage and Disadvantage (IRSAD) and rurality according to the Rural, Remote and Metropolitan Area (RRMA) classification [18] based on each site’s postcodes.

### Costing

Costs were quantified based on reported time spent recruiting and related expenses to support recruitment. Hourly rates for each staff member were calculated based on published salaries for the respective occupations and are listed in Appendix 2 in Supplementary Material. Staff cost varied from $25.15 per hour for administrative staff to $108.31 per hour for medical staff. Thirty percent was added to staff costs to account for overheads. The study coordinator made at least one trip to each of the study sites to support patient recruitment. The total number of trips during the study period was estimated based on records from the clinical trial data. Cost was estimated based on distance travelled (return trip) from the research office of the study coordinator to study site multiplied by the recommended cost rate for each kilometre travelled [19].

### Statistical analysis

The three key outcomes assessed were recruitment efficiency (ratio of screened to randomised), average time used, and cost for each participant randomised. Efficiency of identification (ratio of screened to eligible participants) and conversion to enrolment (ratio of eligible to randomised participants) were also calculated from data available to explore the recruitment steps (e.g. identify patients, screening, consenting) that may be driving recruitment efficiency. To identify the practice-level factors associated with efficient recruitment and lower cost, outcomes were dichotomised (25th percentile vs others) and each practice-level factor was assessed against the outcomes to determine its association.

## Results

Across 25 general practice (GP) study sites, 1968 participants were screened of which 1157 were determined to be eligible. Of these, 721 declined to participate or did not respond, resulting in 299 (15.2%) of initially screened participants ultimately recruited and randomised. The flow of participants in the study is presented in Appendix 3. Table 1 shows the recruitment of participants by study site and results of the key outcomes assessed.

**Table 1:** Recruitment efficiency and recruitment costs

Practice ID	Database screened	Eligible	Randomised	Recruitment efficiency^**a**^	Staff time per patient randomised (hours)	Cost per randomised patient ($)
1	20	15	14	1.43	2.29	167
2	86	64	8	10.75	5	366
3	26	16	16	1.63	4	276
4	31	14	6	5.17	6.5	435
5	113	54	21	5.38	2.48	188
6	123	100	17	7.24	2.76	220
7	22	7	2	11	11	797
8	96	23	10	9.6	3.3	213
9	31	25	9	3.44	1.11	74
10	116	54	22	5.27	3.09	254
11	98	59	8	12.25	4.38	307
12	32	17	16	2	1.75	144
13	66	34	5	13.2	8.4	574
14	98	55	16	6.13	2.63	179
15	15	12	2	7.5	2.5	181
16	49	17	7	7	7.14	472
17	168	121	21	8	0.62	127
18	17	6	2	8.5	4.5	330
19	169	106	30	5.63	1.4	132
20	75	39	6	12.5	5.33	357
21	44	26	12	3.67	2.33	179
22	164	116	28	5.86	2.07	147
23	117	39	8	14.63	3.25	234
24	73	45	7	10.43	3.71	269
25	119	93	6	19.83	5	314
**Mean (SD)**	**78.7 (49.4)**	**46.3 (35.6)**	**12 (7.9)**	**7.9 (4.4)**	**3.9 (2.4)**	**277(160.6)**

^a^Calculated as participants screened/participants randomised

**Fig. 1:**
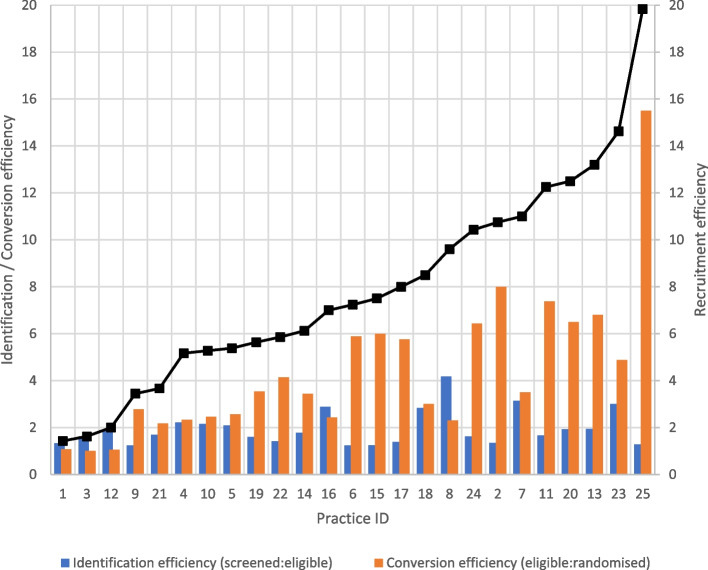
Efficiency of identification and conversion across all study sites. The black line represents the recruitment efficiency

The mean recruitment efficiency (number of participants recruited and randomised relative to numbers screened) was 7.9, and this varied widely across sites from 1.4 to 19.8. A higher conversion to enrolment ratio was observed amongst sites that were less efficient in recruitment (Fig. 1), while the conversion of screened to eligible participants’ ratio (identification) was more consistent across sites. Seven study sites were identified as the 25th percentile most efficient recruitment sites (recruitment efficiency of less than 5.3), and the strongest factor associated with efficiency was assigning medical and nursing staff (instead of practice administrative staff) responsible for identifying potential participants (57.14% vs. 22.2%) (Table 2). Although the results did not show statistical significance (likely due to small sample size), sites that were most efficient comprised of smaller practices and were more likely to be rural locations and in areas of lower SES (Table 2). Other factors positively associated with recruitment efficiency were availability of multiple clinical audit tools and associated training for these tools and a supportive culture for research at site.

**Table 2:** Comparisons by most efficient and lowest cost sites

Characteristic	By recruitment efficiency		By recruitment cost	
	Most efficient^**a**^	Others	Lowest cost^**a**^	Others
	(***n*** = 7)	(***n*** = 18)	(***n*** = 7)	(***n*** = 18)
**Site characteristics**				
Practice size (total GP FTE), mean (SD)	4.39 (2.21)	7.31 (4.59)	7.13 (4.05)	6.24 (4.39)
Rural location	2 (29%)	2 (11%)	2 (29%)	2 (11%)
Socioeconomic index^b^				
1/2	2 (29%)	6 (33%)	4 (57%)	4 (22%)
3	4 (57%)	3 (17%)	1 (14%)	6 (33%)
4/5	1 (14%)	9 (50%)	2 (29%)	8 (44%)
**Clinical audit tools**				
≥ 2 tools available	2 (29%)	1 (6%)	1 (14%)	2 (11%)
Training to use tools	7 (100%)	14 (82%)	7 (100%)	14 (82%)
**Research culture**				
Concurrently involved in other studies	5 (71%)	10 (56%)	5 (71%)	10 (56%)
If other studies were also diabetes-related	1 (20%)	5 (50%)	2 (40%)	4 (40%)
Involved in ≥ 2 studies in last 3 years	4 (57%)	11 (61%)	7 (100%)	8 (44%)
**Site support**				
Nurse/administrative support (very high/high)	7 (100%)	14 (78%)	7 (100%)	14 (78%)
GP support (very high/high)	7 (100%)	14 (78%)	6 (86%)	15 (83%)
**Recruitment support**				
Access to eligibility information (very easy/easy)	7 (100%)	15 (83%)	7 (100%)	15 (83%)
Medical staff (practice nurse/GP) responsible for identifying potential patients	4 (57%)	4 (22%)	2 (29%)	6 (33%)
Practice nurse coordinate contacting patients	6 (86%)	9 (50%)	5 (71%)	10 (56%)
**Study coordinator’s perspective on recruitment**				
Very easy/easy	6 (86%)	11 (61%)	7 (100%)	10 (56%)
Manageable	1 (14%)	2 (11%)	0 (0%)	3 (17%)
Difficult/very difficult	0 (0%)	5 (28%)	0 (0%)	5 (28%)

^a^25th percentile

^b^1/2 most disadvantaged; 4/5 most advantaged

**Fig. 2:**
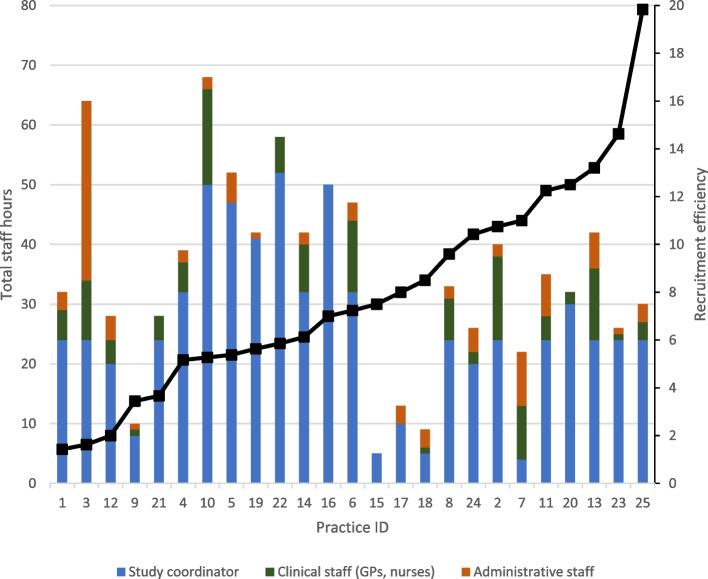
Number of staff hours used for recruitment. The black line represents the recruitment efficiency

The average time used for recruitment was 3.7 h (SD 2.4) per patient randomised. The total staff hours used for recruitment varied across sites, ranging from 5 to 68 hours. Across most sites, the study coordinator contributed to majority of the hours spent on recruitment (Fig. 2). Sites that were more efficient (lower recruitment efficiency ratio) were observed to have used more hours; however, this effect attenuated after adjusting for GP size.

The mean cost per patient randomised was $277 (SD 161), and this varied widely across sites from $74 to $797. The practice sites identified with the 25% lowest recruitment cost (*n* = 7) were significantly more likely to be more experienced in research participation (100% vs. 44.44%). Other factors positively associated with lower recruitment cost were sites in rural areas, of lower SES, being concurrently involved in other studies, reporting a supportive culture for research, and assigning a practice nurse for coordinating contact with participants.

Three sites were identified in the 25th percentile in both recruitment efficiency and lowest cost (Appendix 4 in Supplementary Material), and the strongest factor associated with these sites was assigning medical and nursing staff responsible for identifying potential participants (66.67% vs. 27.27%) and being concurrently involved in other studies and with experience in research participation (100% vs. 54.55%).

## Discussion

Under-recruitment and the high costs of recruiting for clinical trials have been well documented in the literature [1–3]. While most research on trial efficiency is focused on study designs and recruitment strategies, this study is focused on the selection of study sites to optimise recruitment. Using data from an RCT conducted across 25 general practices, this study identified a number of factors that were associated with recruitment and cost efficiency. This included designating appropriate clinical staff (doctors and nurses) to support the recruitment process as well as having a supportive culture for research. Our findings echo those reported by Levett et al. [20] who found that sites that had a supportive and defined research trial structure for recruitment in place consistently had better recruitment rates than those that did not. Having a clear recruitment strategy right at the start and assigned responsibilities is an important enabler to recruitment success [21]. We observe that sites that were more efficient in recruitment dedicated more staff hours to recruitment (Fig. 2) and also found that sites that were concurrently involved in other studies were likely to be more efficient, and it is likely that these sites already have “systems” in place to support the research activities. This suggests that there needs to be adequate resources put in place in ensuring those involved in the trial process are sufficiently set up to facilitate recruitment or that sites should be able to demonstrate fulfilling minimum criteria for recruiting capability prior to being selected.

The proportion of sites in rural locations and low SES areas were observed to be slightly greater in the most efficient and lowest cost category. While our findings regarding rural practices appear to correspond with that reported in a previous study, ASPREE [22], the association between SES and recruitment is less clear. Williams et al [11] reported that GPs located in a high SES area recruited at a lower rate than those in lower SES which contrasts with that reported in ASPREE. Gaining access to research participants in rural or remote areas has been identified as one of the top ten barriers to recruitment [21], and this is being addressed in Australia through the development of tele-trials and rural practice-based research networks such as the PARTNER network [23]. It was observed that the study coordinator made more visits to rural sites (7.5 compared to 4.6 for non-rural sites), and this was likely to have contributed to the recruitment efficiency. Despite the increased number of visits, our results indicate that this seemed to be a reasonable trade off as rural sites remained amongst the most cost efficient. Given the increasing acceptance and application of telehealth and teletrial approaches [24, 25], considerations to include such sites could result in greater efficiencies, with additional benefits of improving equitable access to a more diverse patient population and increasing the generalisability of study findings.

We observe that sites that more successfully converted eligible patients to randomised (Fig. 1) were also more efficient in recruitment. Opportunities to further explore the possible reasons for this through qualitative interviews with the study coordinator and/or site personnel would have proved informative although were not included in the scope of our research design. Despite the growing amount of literature on strategies to improve recruitment rates, researchers lack opportunities to share and learn from the mistakes of others. It is also unknown how many studies of recruitment strategies have not been published due to negative results [10]. Data on the cost and rate of recruitment, risk factors, and challenges faced are not reported as part of the metrics required for clinical trial reporting. Therefore, it is often hard to know the reasons for poor, slow, or failed recruitment such that strategies can be put in place in the trial planning stages to mitigate these challenges. More definitive guidance is needed which is only possible if more data is made available for research or if researchers include evaluations on their recruitment within their trial designs. It would be important to explore opportunities to incorporate the reporting of a minimum criteria that can help trialists plan for successful trials. Collecting data on site characteristics and detailed participant flow such as that collected in this study across other settings (e.g. hospitals) and in other disease areas (e.g. cancer, musculoskeletal diseases) could spur further research in developing a database that can be used as resource for trialists in the planning process. Often such types of research are not funded, but trialists could look for opportunities for building in sub-studies within the trial.

### Limitations

Our study was limited to a comparative descriptive analysis due to the small sample size (*N* = 25) and thus was underpowered to show statistical significance across factors examined. We had hoped to identify factors that were predictive of recruitment and cost efficiency; however, we did not have sufficient power to undertake this analysis. Despite this, the analysis has provided critical insights to improve trial efficiency and helpful indications to trialists on key site characteristics when selecting study sites. These insights provide a number of hypotheses which can be further tested in future studies. We acknowledge that the approach to dichotomise outcomes at the 25th percentile may not be an ideal approach as it resulted in a loss of information and could affect the results are presented in Table 2. However, we considered this a pragmatic decision in helping us identify the key characteristics of top performers amongst the GP sites involved. To test the robustness of our results, we conducted additional analyses to test different cut-off points (20th and 33rd percentile). Overall, the results trended in the same direction (Appendix 5 in Supplementary Material) and are unlikely to change our conclusions. The reporting of time commitment was surveyed at the end of the recruitment period and, therefore, may be subject to inaccuracies in reporting and recall bias. Although this study included a mix of metropolitan and rural GPs, they were from a single Australian state and recruited patients with a specific chronic condition and technology intervention. Therefore, the generalisability of the findings to other settings, disease areas, or intervention focus is unknown. In this study, we captured costs related to staff time required to support recruitment and did not capture cost of tools used to facilitate recruitment. This may have varied across sites and may have contributed to both recruitment efficiency and total cost.

## Conclusion

Despite the small sample size, this study provides helpful indications of site-level characteristics that can help improve the feasibility and efficiency of conducting RCT in GP settings. Characteristics indicative of having appropriate clinical staff (doctors and nurses) supporting the recruitment process and having high levels of support for research and rural practices which often tends to be overlooked were observed to be more efficient in recruiting. Further research to investigate these factors in a wider range of trials will prove useful.

## Supplementary Information

**Additional file 1: Appendix 1.** Questionnaire.

**Additional file 2: Appendix 2.** Staff costs.

**Additional file 3: Appendix 3.** Flow of participants.

**Additional file 4: Appendix 4.** Comparisons of site characteristics between 25th percentile (high recruitment efficiency and low cost) and others.

**Additional file 5: Appendix 5.** Results from robustness tests.

## Data Availability

The datasets used and/or analysed during the current study are available from the corresponding author on reasonable request.

## Abbreviations

ABS: Australian Bureau of Statistics
GP: General practice
IRSAD: Index of Relative Socio-economic Advantage and Disadvantage
SD: Standard deviation
SES: Socioeconomic status
RA: Research assistant
RCT: Randomised controlled trial
RRMA: Rural, Remote and Metropolitan Area
T2D: Type 2 diabetes
