# Supplementary material for: Site-specific factors associated with clinical trial recruitment efficiency in general practice settings: a comparative descriptive analysis
Source: Trials. 2023 Mar 4;24:164. doi: 10.1186/s13063-023-07177-4 (PMC9985191; doi:10.1186/s13063-023-07177-4)
Supplement: Supplementary file 1 — Additional file 1: Appendix 1. Questionnaire. [file 13063_2023_7177_MOESM1_ESM.docx]

**Supplementary Material**

**Appendix 1: Questionnaire**

**PART A: Study site characteristics**

*To be completed by the practice manager or similar at the beginning of research at the practice*

In order to help us understand recruitment and the challenges faced could you please complete the following questions about recruitment in this current practice. This will help us tailor our support and plan resources and training for recruitment.

1. The number of FTE equivalent staff in your practice (please write answers in boxes provided)

| GPs |  |  |
| --- | --- | --- |
| Practice nurses |  |  |
| Support staff (including administrative staff) |  |  |

1. How many other studies is this practice currently involved in? ____________________________
2. Is this practice involved in any other study that involve diabetes patients?

(please circle) Yes No

If yes, how many trials?__________________

1. How many studies has this practice been involved in over the last 3 years?________________
2. What tools does your practice have available to help identify eligible patients for this study? (Please tick all that apply)

| Pen Clinical Assessment tool (PenCAT) |  |
| --- | --- |
| Population Level Analysis and Reporting (POLAR) system |  |
| Other: please specify | |
| Other: please specify | |

1. Do you have someone trained at your practice to use these tools? (please circle)

Yes No Not Applicable

1. From your experience- for this current study please describe your access to the information you will need to determine **eligibility of a cohort** of patients who could be screened? (please circle)

Very easy Easy Manageable Difficult Very difficult

**PART B: Recruitment questionnaire**

*To be completed by the study co-ordinator after they completion of recruitment at each practice*

In order to help us understand recruitment and the challenges faced could you please complete the following questions about recruitment in this current practice. This will help us tailor our support and plan resources and training for recruitment.

1. How easy was it to recruit patients at this practice? (Please circle)

Very easy Easy Manageable Difficult Very difficult

1. How would you describe the nurse/administration support (or willingness to participate and support recruiting) for this current study at this practice?
   1. Very high
   2. High
   3. Mixed
   4. Low
   5. Very low
2. How would you describe the general practitioner support (or willingness to participate and support recruiting) for this current study at this practice?
   1. Very high
   2. High
   3. Mixed
   4. Low
   5. Very low
3. At this practice, who was responsible for identifying the potential study subjects?

|  | Practice nurse |  | Medical staff |
| --- | --- | --- | --- |
|  | Practice manager |  | Other researcher |
|  | Data manager |  | Other administration role |
|  | Research nurse (employed by practice) |  | Other (specify)_________________________ |

1. For this study who was the lead co-ordinating role contact for you at this practice for recruitment?

|  | Practice nurse |  | Medical staff |
| --- | --- | --- | --- |
|  | Practice manager |  | Other researcher |
|  | Data manager |  | Other administration role |
|  | Research nurse (employed by practice) |  | Other (specify)_________________________ |

**PART C: Recruitment questionnaire**

*To be completed by practice manager or similar following the end of recruitment in each practice.*

In order to help us identify the time commitment required from your practice to support patient recruitment in the study please complete the following.

| Total time to recruit these patients (in hours of work in practice) by any of the following who were involved: | **Answer (in hours)** |
| --- | --- |
| 1. Study coordinator |  |
| 1. Practice nurse |  |
| 1. Practice manager |  |
| 1. Data manager |  |
| 1. Research nurse (employed by the practice) |  |
| 1. Medical staff |  |
| 1. Other administrative staff |  |
| 1. Other (please specify)______________________ |  |
