# Supplementary material for: Site-specific factors associated with clinical trial recruitment efficiency in general practice settings: a comparative descriptive analysis
Source: Trials. 2023 Mar 4;24:164. doi: 10.1186/s13063-023-07177-4 (PMC9985191; doi:10.1186/s13063-023-07177-4)
Supplement: Supplementary file 2 — Additional file 2: Appendix 2. Staff costs. [file 13063_2023_7177_MOESM2_ESM.docx]

**Appendix 2: Staff costs**

| **Staff** | **Hourly rate*** | **Source** |
| --- | --- | --- |
| Study coordinator | $49.59 | Sourced online from three open resource websites for average Australian wages:  SalaryExpert (<https://www.salaryexpert.com/>)  Payscale (<https://www.payscale.com/>)  Indeed (<https://au.indeed.com/>) |
| Practice nurse | $41.77 |  |
| Practice manager | $46.00 |  |
| Data manager | $53.22 |  |
| Research nurse (employed by practice) | $45.19 |  |
| Medical staff | $108.31 |  |
| Other administrative staff | $25.15 |  |

*Calculated based on mean wages from all sources and assuming a 38-hour week. Hourly rate = (Annual salary/52)/38
