# Supplementary material for: Site-specific factors associated with clinical trial recruitment efficiency in general practice settings: a comparative descriptive analysis
Source: Trials. 2023 Mar 4;24:164. doi: 10.1186/s13063-023-07177-4 (PMC9985191; doi:10.1186/s13063-023-07177-4)
Supplement: Supplementary file 3 — Additional file 3: Appendix 3. Flow of participants. [file 13063_2023_7177_MOESM3_ESM.docx]

**Appendix 3: Flow of participants**

Assessed for eligibility

(n=1968)*

Eligible participants

(n=1157, 59%)*

Randomised and recruited

(n=299, 26%)

Ineligible (n=811)*

Declined to participate or did not respond (n=721)

* Data presented in this study varied slightly from that presented in the clinical trial outcome paper due to transcription error. These corrections did not affect the clinical trial analysis and outcomes.
