# Supplementary material for: Site-specific factors associated with clinical trial recruitment efficiency in general practice settings: a comparative descriptive analysis
Source: Trials. 2023 Mar 4;24:164. doi: 10.1186/s13063-023-07177-4 (PMC9985191; doi:10.1186/s13063-023-07177-4)
Supplement: Supplementary file 4 — Additional file 4: Appendix 4. Comparisons of site characteristics between 25th percentile (high recruitment efficiency and low cost) and others. [file 13063_2023_7177_MOESM4_ESM.docx]

**Appendix 4: Comparisons of site characteristics between 25^th^ percentile (high recruitment efficiency and low cost) and others**

| **Characteristics** | **Most efficient**  **(n=3)** | **Others**  **(n=22)** |
| --- | --- | --- |
| **Site characteristics** |  |  |
| Practice size (Total GP FTE), mean (SD) | 4.57 (1.40) | 6.75 (4.44) |
| Rural location | 0 (0%) | 4 (19%) |
| Socioeconomic index | 1 (33%) | 7 (32%) |
| 1/2 (most disadvantaged) | 1 (33%) | 6 (27%) |
| 3 | 1 (33%) | 9 (41%) |
| 4/5 (most advantaged) |  |  |
| **Clinical audit tools** |  |  |
| ≥ 2 tools available | 1 (33%) | 2 (9%) |
| Training to use tools | 3 (100%) | 18 (86%) |
| **Research culture** |  |  |
| Concurrently involved in other studies | 3 (100%) | 12 (55%) |
| If other studies were also diabetes-related | 1 (33%) | 5 (42%) |
| Involved in ≥2 studies in last 3 years | 3 (100%) | 12 (55%) |
| **Site support** |  |  |
| Nurse/administrative support (Very high/high) | 3 (100%) | 18 (82%) |
| GP support (Very high/high) | 3 (100%) | 18 (82%) |
| **Recruitment support** |  |  |
| Access to eligibility information (Very easy/easy) | 3 (100%) | 19 (86%) |
| Medical staff (practice nurse/GP) responsible for identifying potential patients | 2 (67%) | 6 (27%) |
| Practice nurse co-ordinate contacting patients | 2 (67%) | 13 (59%) |
| **Study coordinator’s perspective on recruitment** |  |  |
| Very easy/Easy | 100 | 14 (64%) |
| Manageable | 0 (0%) | 3 (14%) |
| Difficult / Very difficult | 0 (0%) | 5 (23%) |
