# Supplementary material for: Site-specific factors associated with clinical trial recruitment efficiency in general practice settings: a comparative descriptive analysis
Source: Trials. 2023 Mar 4;24:164. doi: 10.1186/s13063-023-07177-4 (PMC9985191; doi:10.1186/s13063-023-07177-4)
Supplement: Supplementary file 5 — Additional file 5: Appendix 5. Results from robustness tests. [file 13063_2023_7177_MOESM5_ESM.docx]

**Appendix 5: Results from robustness tests**

**Comparisons of site characteristics between 20^th^ percentile (high recruitment efficiency and low cost) and others**

| **Characteristic** | **By recruitment efficiency** | | **By recruitment cost** | |
| --- | --- | --- | --- | --- |
|  | **Most efficient ^a^** | **Others** | **Lowest cost ^a^** | **Others** |
|  | **(n=5)** | **(n=20)** | **(n=5)** | **(n=20)** |
| **Site characteristics** |  |  |  |  |
| Practice size (Total GP FTE), mean (SD) | 4.04 (1.24) | 7.1 (4.51) | 7.24 (4.89) | 6.3 (4.18) |
| Rural location | 1 (20%) | 3 (15%) | 2 (40%) | 2 (10%) |
| Socioeconomic index^b^ |  |  |  |  |
| 1/2 | 1 (20%) | 7 (35%) | 4 (80%) | 4 (20%) |
| 3 | 3 (60%) | 4 (20%) | 0 (0%) | 7 (35%) |
| 4/5 | 1 (20%) | 9 (45%) | 1 (20%) | 9 (45%) |
| **Clinical audit tools** |  |  |  |  |
| ≥ 2 tools available | 1 (20%) | 2 (10%) | 1 (20%) | 2 (10%) |
| Training to use tools | 5 (100%) | 16 (84%) | 5 (100%) | 16 (84%) |
| **Research culture** |  |  |  |  |
| Concurrently involved in other studies | 4 (80%) | 11 (55%) | 4 (80%) | 11 (55%) |
| If other studies were also diabetes-related | 1 (25%) | 5 (45%) | 1 (25%) | 5 (45%) |
| Involved in ≥2 studies in last 3 years | 4 (80%) | 11 (55%) | 5 (100%) | 10 (50%) |
| **Site support** |  |  |  |  |
| Nurse/administrative support (Very high/high) | 5 (100%) | 16 (80%) | 5 (100%) | 16 (80%) |
| GP support (Very high/high) | 5 (100%) | 16 (80%) | 4 (80%) | 17 (85%) |
| **Recruitment support** |  |  |  |  |
| Access to eligibility information (Very easy/easy) | 5 (100%) | 17 (85%) | 5 (100%) | 17 (85%) |
| Medical staff (practice nurse/GP) responsible for identifying potential patients | 4 (80%) | 4 (20%) | 1 (20%) | 7 (35%) |
| Practice nurse co-ordinate contacting patients | 4 (80%) | 11 (55%) | 4 (80%) | 11 (55%) |
| **Study coordinator’s perspective on recruitment** |  |  |  |  |
| Very easy/Easy | 4 (80%) | 13 (65%) | 5 (100%) | 12 (68%) |
| Manageable | 1 (20%) | 2 (10%) | 0 (0%) | 3 (12%) |
| Difficult / Very difficult | 0 (0%) | 5 (25%) | 0 (0%) | 5 (25%) |

^a^ 20^th^ percentile

^b^ 1/2 most disadvantaged; 4/5 most advantaged

**Comparisons of site characteristics between 33^rd^ percentile (high recruitment efficiency and low cost) and others**

| **Characteristic** | **By recruitment efficiency** | | **By recruitment cost** | |
| --- | --- | --- | --- | --- |
|  | **Most efficient ^a^** | **Others** | **Lowest cost ^a^** | **Others** |
|  | **(n=9)** | **(n=16)** | **(n=9)** | **(n=16)** |
| **Site characteristics** |  |  |  |  |
| Practice size (Total GP FTE), mean (SD) | 5.72 (3.35) | 6.92 (4.70) | 7.04 (3.87) | 6.18 (4.51) |
| Rural location | 3 (33%) | 1 (6%) | 3 (33%) | 1 (6%) |
| Socioeconomic index^b^ |  |  |  |  |
| 1/2 | 4 (44%) | 4 (25%) | 4 (44%) | 4 (25%) |
| 3 | 4 (44%) | 3 (19%) | 2 (22%) | 5 (31%) |
| 4/5 | 1 (11%) | 9 (56%) | 3 (33%) | 7 (44%) |
| **Clinical audit tools** |  |  |  |  |
| ≥ 2 tools available | 3 (33%) | 0 (0%) | 1 (11%) | 2 (13%) |
| Training to use tools | 9 (100%) | 12 (80%) | 9 (100%) | 12 (80%) |
| **Research culture** |  |  |  |  |
| Concurrently involved in other studies | 6 (67%) | 9 (56%) | 7 (78%) | 8 (50%) |
| If other studies were also diabetes-related | 2 (33%) | 4 (44%) | 2 (29%) | 4 (50%) |
| Involved in ≥2 studies in last 3 years | 6 (67%) | 9 (56%) | 9 (100%) | 6 (38%) |
| **Site support** |  |  |  |  |
| Nurse/administrative support (Very high/high) | 9 (100%) | 12 (75%) | 8 (89%) | 13 (81%) |
| GP support (Very high/high) | 8 (89%) | 13 (81%) | 7 (78%) | 14 (88%) |
| **Recruitment support** |  |  |  |  |
| Access to eligibility information (Very easy/easy) | 8 (89%) | 14 (88%) | 9 (100%) | 13 (81%) |
| Medical staff (practice nurse/GP) responsible for identifying potential patients | 4 (44%) | 4 (25%) | 3 (33%) | 5 (31%) |
| Practice nurse co-ordinate contacting patients | 7 (78%) | 8 (50%) | 6 (67%) | 9 (56%) |
| **Study coordinator’s perspective on recruitment** |  |  |  |  |
| Very easy/Easy | 8 (89%) | 9 (56%) | 8 (89%) | 9 (56%) |
| Manageable | 1 (11%) | 2 (13%) | 0 (0%) | 3 (19%) |
| Difficult / Very difficult | 0 (0%) | 5 (21%) | 1 (11%) | 4 (25%) |

^a^ 33^rd^ percentile

^b^ 1/2 most disadvantaged; 4/5 most advantaged
